# Case Report: Masquerading doughnut: a case of misdiagnosed ileocecal tumor-induced intussusception

**DOI:** 10.3389/fonc.2025.1716593

**Published:** 2025-12-19

**Authors:** Chuchu Xu, Renjun Zhu, Qingfeng Dai, Guangen Xu, Guolin Zhang

**Affiliations:** 1 Department of Gastrointestinal Surgery, Shaoxing People’s Hospital (The First Hospital of Shaoxing University), Shaoxing, Zhejiang, China; 2 School of Medicine, Shaoxing University, Shaoxing, Zhejiang, China; 3 Department of Emergency, Shaoxing People’s Hospital (The First Hospital of Shaoxing University), Shaoxing, Zhejiang, China; 4 Zhijiang College, Zhejiang University of Technology, Shaoxing, Zhejiang, China

**Keywords:** colorectal cancer (CRC), diagnostic challenges, intussusception, Occam’s razor principle, young adults

## Abstract

**Background:**

Intussusception in adults is rare and is often associated with an underlying pathology such as tumors. Its coexistence with colorectal cancer (CRC) in young patients presents unique diagnostic challenges as imaging may suggest separate lesions rather than a single malignancy.

**Case presentation:**

A 21-year-old man presented with worsening right upper and central abdominal pain. The contrast-enhanced abdominal CT suggested that the intussusception at the ileocecal region might be caused by a lipoma and revealed a separate mass in the transverse colon. Emergency laparoscopic exploration and subsequent open laparotomy confirmed a 4.0-cm × 5.0-cm cauliflower-like adenocarcinoma originating from the ileocecal region, which had caused the intussusception and mimicked separate pathologies on imaging.

**Conclusions:**

This case highlights the diagnostic complexity of synchronous intestinal lesions in young patients. It underscores the need for a heightened suspicion of an underlying malignancy when encountering intussusception and emphasizes the limitations of imaging in accurately characterizing the complex pathology of the bowel.

## Background/introduction

Colorectal cancer (CRC) in young adults under 25 years of age accounts for less than 1% of all cases, with only 0.08 cases per 100,000 population reported in surveillance studies (1). While intussusception is a recognized but rare initial manifestation (occurring in 0.3%–0.5% of colorectal malignancies) (2), the coexistence of intussusception and synchronous colonic mass poses unique diagnostic challenges. Such presentations are frequently misattributed to benign etiologies such as lipomas or inflammatory lesions, particularly when the lesions appear anatomically indistinct on imaging (3).

This case highlights a critical diagnostic pitfall where ileocecal intussusception and a transverse colon mass were initially interpreted as separate pathological entities. Preoperative CT findings suggested two discrete lesions: 1) ileocecal intussusception with fatty infiltration and 2) a transverse colon wall thickening suspicious for primary neoplasm. Intraoperative exploration unexpectedly revealed these “two lesions” to represent a single adenocarcinoma originating from the ileocecal region, which had progressively intussuscepted into the transverse colon while dragging adjacent mesenteric adipose tissue. This anatomical distortion created the illusion of synchronous pathologies, misleading both radiological and initial surgical assessments.

This case underscores three critical clinical lessons:

1. Youth does not preclude malignancy: 13.6% of young-onset CRC presents with atypical symptoms masquerading as benign conditions (4).
2. Intussusception complexity: Tumor-led intussusception may create “pseudo-lesions” at distant sites through mechanical traction.
3. Limitations of imaging: The accuracy of CT for distinguishing tumor-induced intussusception components drops to a minimum of 58% when there is complex mesenteric involvement (5).

Despite eventual appropriate management with right hemicolectomy, this diagnostic journey emphasizes the need for a heightened suspicion of malignant continuity when confronting anatomically discordant lesions in young patients.

## Case presentation

A 21-year-old male patient presented to our institution with a 3-day history of progressively worsening right upper and central abdominal pain. The patient denied identifiable triggers or associated gastrointestinal symptoms, including abdominal distension, vomiting, diarrhea, or constipation. No prior abdominal surgical history was reported.

The initial assessment revealed stable vital signs except for an elevated blood pressure (143/95 mmHg). Physical examination demonstrated mild abdominal distension with right-sided tenderness and absent rebound tenderness or palpable masses. Laboratory investigations showed hemoglobin of 142 g/L with microcytic hypochromic parameters [mean corpuscular volume (MCV) = 73.8 fl (normal range, 82.0–100.0) and mean corpuscular hemoglobin (MCH) = 24.9 pg (normal range, 27.0–34.0)]. Other parameters, including the white blood cell count, C-reactive protein, serum electrolytes, hepatic/renal function, and gastrointestinal tumor markers, remained within normal ranges.

**Figure 1:**
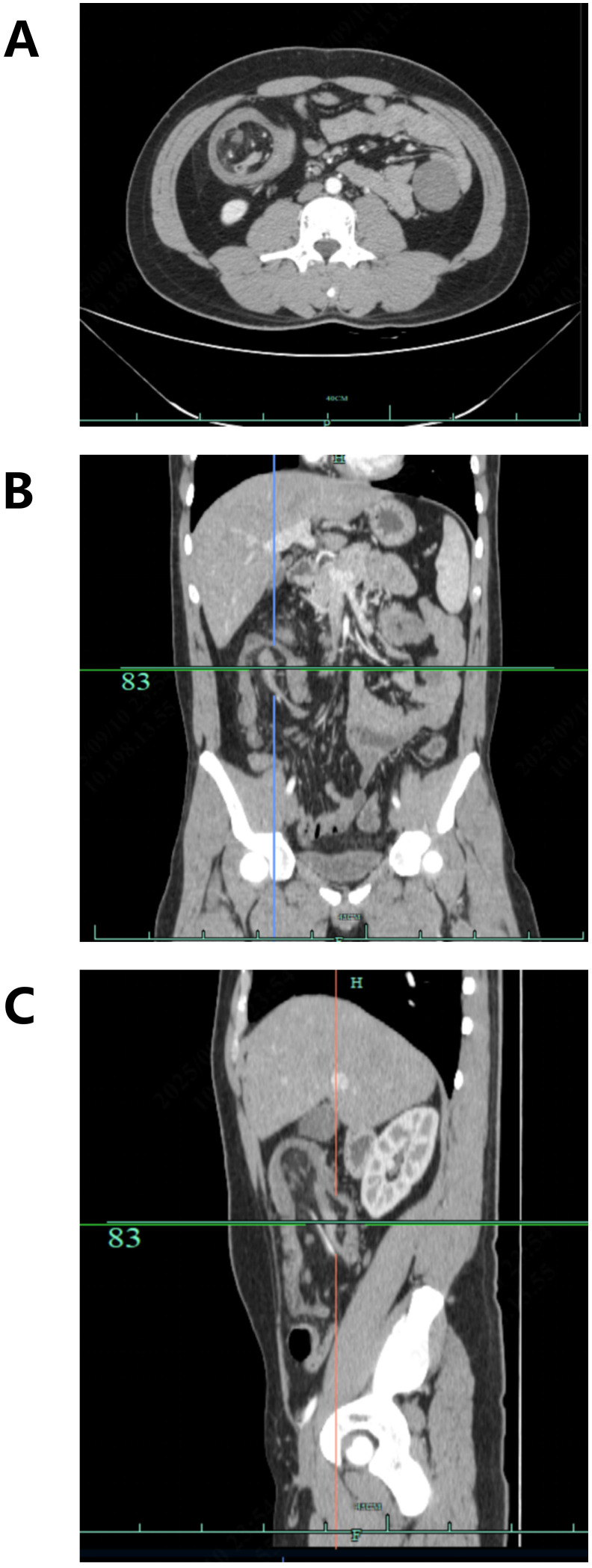
Axial **(A)**, coronal **(B)**, and sagittal **(C)** contrast-enhanced arterial-phase abdominal CT images demonstrating ileocecal intussusception in the affected patient.

**Figure 2:**
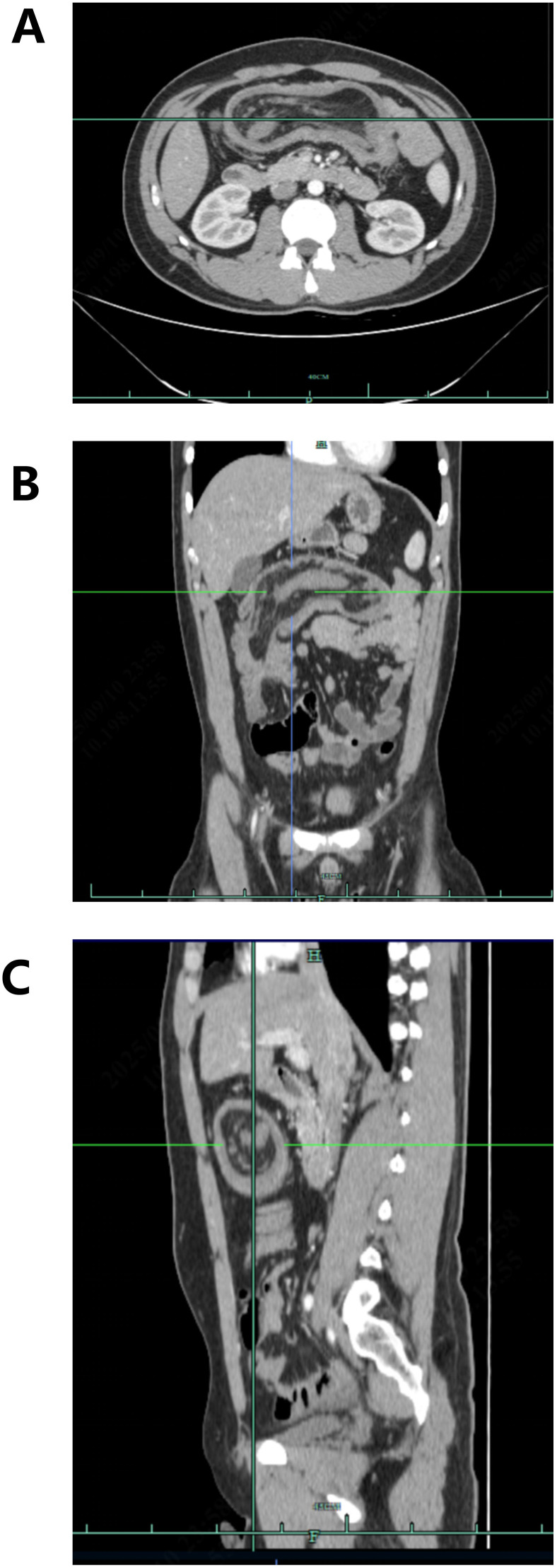
Axial **(A)**, coronal **(B)**, and sagittal **(C)** contrast-enhanced arterial-phase abdominal CT images revealing thickening of the transverse colon wall, raising suspicion of a primary tumor.

Contrast-enhanced abdominal CT revealed dilation of the right colonic lumen with intraluminal entrapment of the small bowel loops and fatty tissue, consistent with intussusception. In addition, there was thickening of the proximal transverse colon wall, raising suspicion of a primary tumor in the proximal transverse colon. Minimal pelvic effusion was also noted (**Figures 1**, **2**).

**Figure 3:**
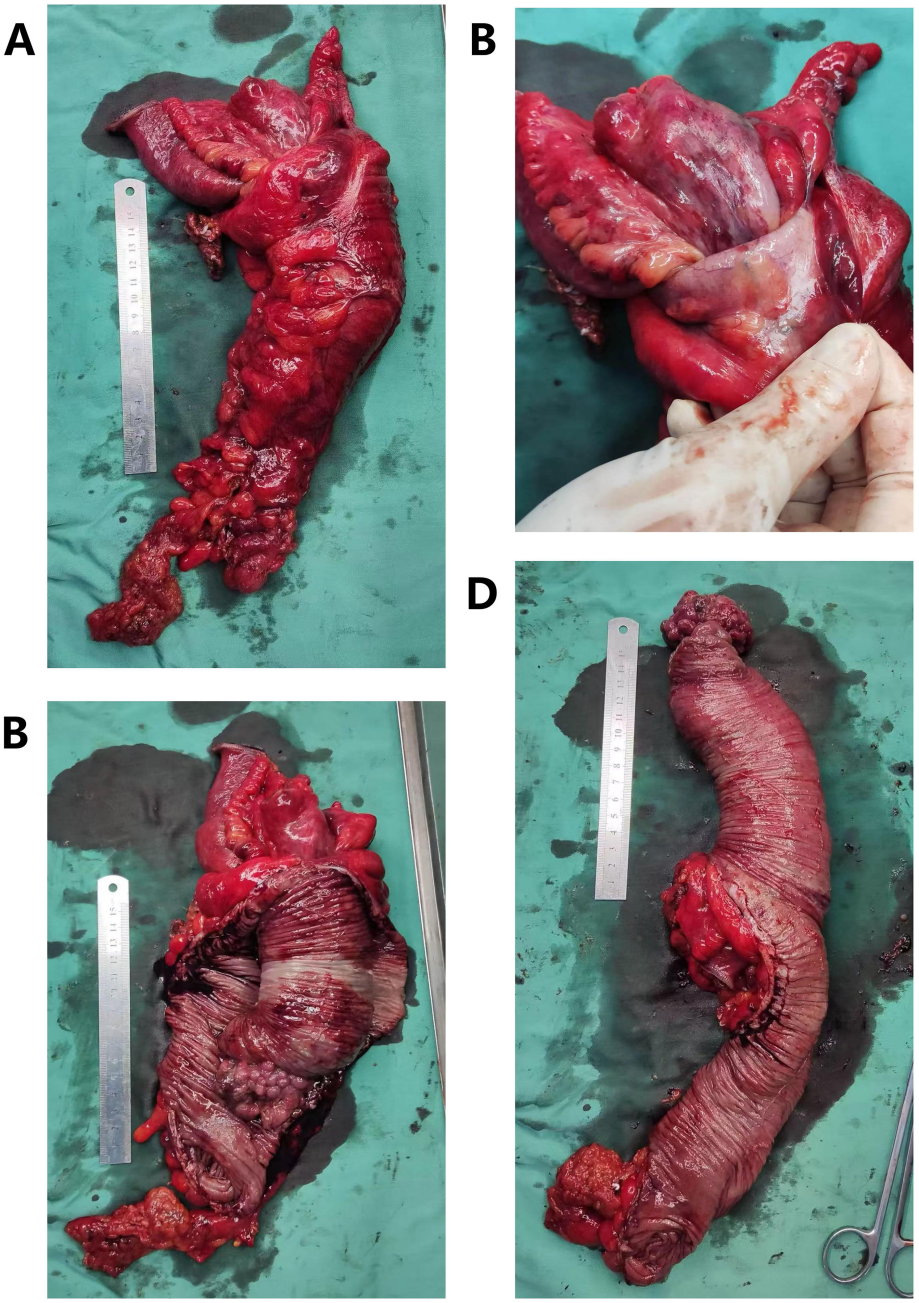
Gross pathological findings following right hemicolectomy. **(A)** Resected right hemicolectomy specimen showing intussusception of the ileocecal region into the colon. A 4-cm × 5-cm mass is palpable in the transverse colon. **(B)** The ileocecal region is intussuscepted into the colon and cannot be reduced by manual traction. **(C)** After opening the colon, the tumor is located in the ileocecal region (more specifically, at the initial segment of the colon, adjacent to the ileocecal valve), measuring 5.5 cm × 4.5 cm × 4 cm. The gross morphology is protrusive. **(D)** After full dissection of the colon and the intussuscepted small intestine, the measurements are as follows: colon length, 22 cm; diameter, 9 cm; ileum length, 25 cm; diameter, 10 cm.

**Figure 4:**
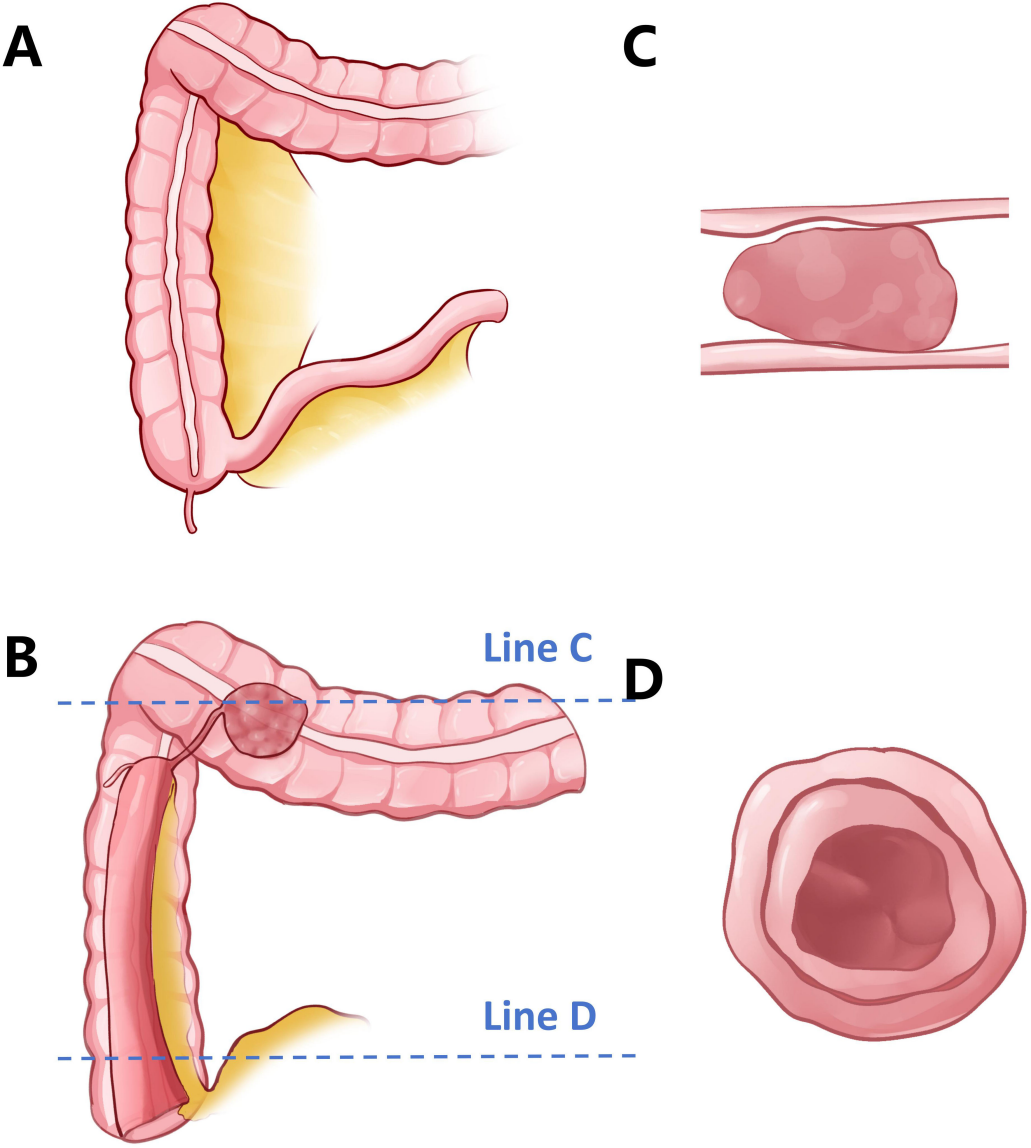
Schematic illustration of the intussusception in this case. **(A)** Normal anatomy of the ileocecal region and the right colon. **(B)** In this patient, a 4-cm × 5-cm mass is present in the ileocecal region. The entire ileocecal segment has intussuscepted into the ascending colon, resulting in the mass being positioned within the lumen of the transverse colon at the time of presentation. **(C)** Cross-sectional view along *line C* in **(B)**. The tumor appears to originate from the transverse colon, mimicking a primary lesion. **(D)** Cross-sectional view along *line D* in **(B)**. The ileocecal region exhibits a “target sign,” a “bulls-eye sign,” or sausage-shaped lesions, consistent with classic imaging features of intussusception.

Given the suspicion of intussusception caused by a lipoma and the presence of a primary tumor in the transverse colon, we proceeded with an emergency laparoscopic exploration. Intraoperatively, we found irreducible ileocecal intussusception into the ascending colon, accompanied by a distinct mass in the transverse colon. Upon conversion to open laparotomy, the persistent ileocecal intussusception was confirmed, which involved the adjacent ascending colon and mesentery. The mass in the transverse colon was identified as a 4.0-cm × 5.0-cm cauliflower-like protrusion originating from the ileocecal region (**Figure 3**). In other words, the mass in the transverse colon observed on the preoperative contrast-enhanced abdominal CT was actually the primary tumor in the ileocecal region that had caused the ileocecal intussusception (**Figure 4**)!

Definitive surgical management involved right hemicolectomy. Histopathological examination confirmed a moderately differentiated adenocarcinoma [T2N0M0, stage I according to the American Joint Committee on Cancer (AJCC), 8th edition] with focal infiltration into the muscularis propria. Molecular profiling demonstrated wild-type status for *KRAS*, *NRAS*, *BRAF*, and *PIK3CA* mutations.

The patient achieved an uneventful postoperative recovery and was discharged 2 weeks following surgery. No adjuvant radiotherapy, chemotherapy, targeted therapy, or immunotherapy was administered during the perioperative period. During the 18-month follow-up assessments via telephone consultation and clinical surveillance, the patient reported sustained complete resolution of his abdominal symptoms. Serial monitoring through contrast-enhanced abdominal CT scans, ultrasound, and colonoscopy and tumor marker evaluations demonstrated no evidence of tumor recurrence, with all relevant biomarkers remaining within the normal ranges.

## Discussion

Intussusception, a common acute abdomen in infants under 2 years of age (rare in adults), is a strangulating intestinal obstruction caused by the invagination of an intestinal segment with its mesentery into an adjacent intestinal lumen, resulting in an obstructed intestinal content passage (6). Ultrasound is the preferred imaging modality for acute intussusception in the emergency department, featuring noninvasiveness, no radiation exposure, and real-time dynamic visualization with a diagnostic accuracy exceeding 95%. For uncomplicated intussusception in children, ultrasound-guided hydrostatic reduction (USG-HR) is the standard and internationally preferred non-surgical treatment modality (7).

As a general rule, intussusception in patients older than 6 years of age is often pathological rather than physiological. Common etiologies typically include Meckel’s diverticulum, intestinal polyps, or, in some cases, tumors (2). For this reason, USG-HR is not the standard treatment for adult intussusception. Surgical intervention remains the preferred and standard approach to avoid delaying the diagnosis and treatment of malignant lesions. USG-HR may only be considered in extremely rare special cases (e.g., transient, self-limiting intussusception without identifiable precipitating factors confirmed by imaging).

### Diagnostic challenges in adult intussusception

Adult intussusception presents significant diagnostic challenges, representing only 1% of bowel obstructions and 5% of all intussusception cases, with malignancy found in 48%–78% of the colonic subtypes (8). Although classically associated with abdominal pain, a palpable mass, and bloody stools, this triad appears in just 25.6% of patients (9). Our patient’s case illustrates the diagnostic dilemma, presenting with 3 months of intermittent right lower quadrant discomfort initially attributed to dietary factors—a pattern consistent with studies showing that 63% of adult intussusception cases exhibit nonspecific symptoms, leading to a delay in the cancer diagnosis (10). Generally speaking, intussusception typically appears on abdominal CT or ultrasound as a “target sign,” a “bulls-eye sign,” or sausage-shaped lesions, presenting as a concentric hyperdense double ring. More vividly, it resembles a doughnut. However, such changes are not absolute. It is precisely for this reason that the efficacy of abdominal CT and ultrasound in diagnosing intussusception is limited (11).

### Epidemiologic paradox in young-onset CRC

Recent surveillance data have indicated that while the incidence of CRC peaks between ages 65 and 74 years, a notable 3.06% of cases now occur in individuals under 50 years old (12). Importantly, 50% of young patients with CRC do not have familial cancer syndromes or known pathogenic mutations such as *APC* or *MLH1* (13). Our patient’s case illustrates this sporadic pattern well, as he had no family history of cancer, he tested negative for *KRAS*, *NRAS*, *BRAF*, and *PIK3CA* mutations, and he showed no signs of inflammatory bowel disease. The initial dismissal of malignancy despite CT evidence of a colonic mass highlights a concerning cognitive bias—studies show that surgeons are less likely to suspect CRC in young patients presenting with acute abdominal symptoms (14). This underscores the need for greater awareness of CRC in younger populations.

### Intraoperative decision-making and pathophysiological insights

The extensive ileocecal intussusception with a tumor in our case presented unique technical challenges, differing significantly from pediatric cases where USG-HR is standard (7). Current guidelines strongly discourage aggressive manipulation in adults with suspected malignancy due to three major risks: 1) tumor seeding from longitudinal traction forces, which can spread malignant cells along the serosal surface (observed in CRC-related intussusception cases) (15); 2) ischemic compromise from prolonged intussusception (>48 h), which increases mural edema and the risk of rupture during manipulation—particularly concerning given our patient’s 72-h symptom duration; and 3) diagnostic interference, as reduction obscures the lead point, complicating histopathological assessment.

The transverse colon “mass” we discovered illustrated a biomechanical paradox of tumor progression, with histopathological mapping showing that the adenocarcinoma originated at the ileocecal region, growing intraluminally while being propelled antegrade by peristaltic forces. This created a “telescoping tumor complex,” where the neoplasm acted as both a lead point and an intussusceptum, dragging the adjacent mesentery to mimic a secondary lesion—a phenomenon explaining why the right-sided CRC intussusceptions showed pseudo-synchronous lesions on imaging.

Our choice to proceed with en bloc right hemicolectomy despite diagnostic uncertainty aligns with oncological principles, as recent meta-analyses have shown comparable 5-year survival rates between emergency CRC resections (74.2%) and elective surgeries (76.8%) when R0 margins are achieved (16).

### Applying Occam’s razor to complex clinical problems

While our definitive right hemicolectomy followed standard oncological principles to achieve R0 margins and complete mesocolic excision (CME) (17), this case highlights an important challenge to the common assumption of dual pathology often seen in emergency surgeries. Studies have shown that, when faced with unusual findings such as multiple masses during surgery, surgeons tend to consider multiple diagnoses—a decision-making pattern known as “diagnostic pluralism.” (18, 19) This approach, which is influenced by time constraints and confirmation bias, leads to unnecessary extended resections in colorectal emergency cases, even when not supported by pathology results.

In clinical medicine, Occam’s razor means choosing the simplest explanation for symptoms. It advises doctors to consider the most common causes first, avoiding unnecessary complexity. Our experience underscores the continued importance of Occam’s razor—the principle that the simplest explanation is often the correct one—in surgical diagnosis. What initially appeared to be a separate mass in the transverse colon turned out to be mesenteric fat pulled by the tumor, illustrating how a complex tumor behavior can mimic multiple conditions. Research indicates that larger tumors (≥3 cm) can create enough pressure inside the intestine to cause telescoping (intussusception) and trap nearby fat, leading to false signs of additional tumors on imaging in nearly a third of cases (20). This supports the “unified traction theory,” which explains how a primary tumor can mechanically distort nearby tissues, creating a misleading appearance (21).

### Reappraising preoperative imaging nuances

Postoperative CT reevaluation revealed previously missed subtle tumor signs, including eccentric bowel wall thickening and disrupted mucosal enhancement patterns. These findings align with research indicating that the initial misinterpretations stem from insufficient attention to secondary CT markers, such as mesenteric fat stranding and asymmetric lymphadenopathy, rather than technical limitations (22). Despite the 58%–100% accuracy of CT in differentiating the etiology of intussusception (23), our clinical experience highlights the critical need for systematic multiplanar analysis, especially in younger patient populations where malignancy often remains a secondary diagnostic consideration. The case underscores how meticulous reevaluation of the imaging findings can uncover significant diagnostic clues that were initially overlooked.

## Data Availability

The original contributions presented in the study are included in the article/supplementary material. Further inquiries can be directed to the corresponding author.

## References

[1] Siegel RL Wagle NS Cercek A Smith RA Jemal A . Colorectal cancer statistics, 2023. CA Cancer J Clin. (2023) 73:233–54. doi: 10.3322/caac.21772, PMID: 36856579

[2] Marinis A Yiallourou A Samanides L Dafnios N Anastasopoulos G Vassiliou I . Intussusception of the bowel in adults: A review. World J Gastroenterol. (2009) 15:407. doi: 10.3748/wjg.15.407, PMID: 19152443 PMC2653360

[3] Kim YH Blake MA Harisinghani MG Archer-Arroyo K Hahn PF Pitman MB . Adult intestinal intussusception: CT appearances and identification of a causative lead point. RadioGraphics. (2006) 26:733–44. doi: 10.1148/rg.263055100, PMID: 16702451

[4] Patel SG Ahnen DJ . Colorectal cancer in the young. Curr Gastroenterol Rep. (2018) 20:15. doi: 10.1007/s11894-018-0618-9, PMID: 29616330

[5] Yakan S Calıskan C Makay O Deneclı AG Korkut MA . Intussusception in adults: Clinical characteristics, diagnosis and operative strategies. World J Gastroenterol. (2009) 15:1985. doi: 10.3748/wjg.15.1985, PMID: 19399931 PMC2675089

[6] DiFiore JW . Intussusception. Semin Pediatr Surg. (1999) 8:214–20. doi: 10.1016/S1055-8586(99)70029-6, PMID: 10573432

[7] Khong PL Peh WC Lam CH Chan KL Cheng W Lam WW . Ultrasound-guided hydrostatic reduction of childhood intussusception: technique and demonstration. RadioGraphics. (2000) 20:e1–1. doi: 10.1148/radiographics.20.5.g00see11, PMID: 10992040

[8] Lianos G Xeropotamos N Bali C Baltoggiannis G Ignatiadou E . Adult bowel intussusception: presentation, location, etiology, diagnosis and treatment. Il G Chir. (2013) 34:280–3., PMID: 24629817 PMC3926485

[9] Das MK Arora NK Gupta B Sharan A Kameswari K Padmalatha P . Intussusception in children aged under two years in India: Retrospective surveillance at nineteen tertiary care hospitals. Vaccine. (2020) 38:6849–57. doi: 10.1016/j.vaccine.2020.04.059, PMID: 32553492 PMC7528221

[10] Honjo H Mike M Kusanagi H Kano N . Adult intussusception: a retrospective review. World J Surg. (2015) 39:134–8. doi: 10.1007/s00268-014-2759-9, PMID: 25192846 PMC4273082

[11] Spaander MCW Zauber AG Syngal S Blaser MJ Sung JJ You YN . Young-onset colorectal cancer. Nat Rev Dis Primer. (2023) 9:21. doi: 10.1038/s41572-023-00432-7, PMID: 37105987 PMC10589420

[12] Shao B Zhu M Shen K Luo L Du P Li J . Disease burden of total and early-onset colorectal cancer in China from 1990 to 2019 and predictions of cancer incidence and mortality. Clin Epidemiol. (2023) 15:151–63. doi: 10.2147/CLEP.S391058, PMID: 36755975 PMC9900241

[13] Mauri G Sartore-Bianchi A Russo A Marsoni S Bardelli A Siena S . Early-onset colorectal cancer in young individuals. Mol Oncol. (2019) 13:109–31. doi: 10.1002/1878-0261.12417, PMID: 30520562 PMC6360363

[14] Law JH Koh FH Tan KK . Young colorectal cancer patients often present too late. Int J Colorect Dis. (2017) 32:1165–9. doi: 10.1007/s00384-017-2837-1, PMID: 28523473

[15] Kopanska KS Alcheikh Y Staneva R Vignjevic D Betz T . Tensile forces originating from cancer spheroids facilitate tumor invasion. Engler AJ Ed PloS One. (2016) 11:e0156442. doi: 10.1371/journal.pone.0156442, PMID: 27271249 PMC4896628

[16] Zhou H Jin Y Wang J Chen G Chen J Yu S . Comparison of short-term surgical outcomes and long-term survival between emergency and elective surgery for colorectal cancer: a systematic review and meta-analysis. Int J Colorect Dis. (2023) 38:41. doi: 10.1007/s00384-023-04334-8, PMID: 36790519

[17] Ueno H Hase K Shiomi A Shiozawa M Ito M Sato T . Optimal bowel resection margin in colon cancer surgery: prospective multicentre cohort study with lymph node and feeding artery mapping. Lancet Reg Health - West Pac. (2023) 33:100680. doi: 10.1016/j.lanwpc.2022.100680, PMID: 37181532 PMC10166781

[18] Aberegg SK Poole BR Locke BW . Hickam’s dictum: an analysis of multiple diagnoses. J Gen Intern Med. (2024) doi: 10.1007/s11606-024-09120-y, PMID: 39467949

[19] Aftab A Banicki K Ruffalo ML Frances A . Psychiatric diagnosis: A clinical guide to navigating diagnostic pluralism. J Nerv Ment Dis. (2024) 212:445–54. doi: 10.1097/NMD.0000000000001791, PMID: 39079000

[20] Brogna B Maccioni F Sgambato D Capuano F Iovine L Guarino S . The many faces of intestinal tumors in adults, including the primary role of CT imaging in emergencies and the important role of cross-sectional imaging: A pictorial review. Healthcare. (2025) 13:1071. doi: 10.3390/healthcare13091071, PMID: 40361849 PMC12071709

[21] Xin Y Li K Huang M Liang C Siemann D Wu L . Biophysics in tumor growth and progression: from single mechano-sensitive molecules to mechanomedicine. Oncogene. (2023) 42:3457–90. doi: 10.1038/s41388-023-02844-x, PMID: 37864030 PMC10656290

[22] Marsicovetere P Ivatury S White B Holubar S . Intestinal intussusception: etiology, diagnosis, and treatment. Clin Colon Rectal Surg. (2016) 30:030–9. doi: 10.1055/s-0036-1593429, PMID: 28144210 PMC5179276

[23] Valentini V Buquicchio GL Galluzzo M Ianniello S Di Grezia G Ambrosio R . Intussusception in adults: the role of MDCT in the identification of the site and cause of obstruction. Gastroenterol Res Pract. (2016) 2016:1–10. doi: 10.1155/2016/5623718, PMID: 26819606 PMC4706914

